# A Greenhouse Test to Explore and Evaluate Light-Emitting Diode (LED) Insect Traps in the Monitoring and Control of *Trialeurodes vaporariorum*

**DOI:** 10.3390/insects11020094

**Published:** 2020-02-01

**Authors:** Jihong Zhang, Huyin Li, Maorong Liu, Huan Zhang, Hai Sun, Hongtuo Wang, Lin Miao, Miaomiao Li, Ruihao Shu, Qilian Qin

**Affiliations:** 1 State Key Laboratory of Integrated Management of Pest Insects and Rodents, Institute of Zoology, Chinese Academy of Sciences, 1 Beichen West Road, Beijing 100101, China; zhangjh@ioz.ac.cn (J.Z.); zhanghuan@ioz.ac.cn (H.Z.); wanght@ioz.ac.cn (H.W.); miaolin@ioz.ac.cn (L.M.); ytqxlmm@163.com (M.L.); shuruihao@ioz.ac.cn (R.S.); 2 Harmony Farm, Erdos 017299, China; lihuyin1982@163.com (H.L.); sh1013089443@163.com (H.S.); 3 Plant Protection and Quarantine Station of Erdos, Erdos 017000, China; erdoszbz@sina.com; 4 College of Life Sciences, University of Chinese Academy of Sciences, No. 19(A) Yuquan Road, Beijing 100049, China

**Keywords:** attractive light, environment light intensity, field evaluation, light trap, phototaxis

## Abstract

Population control of small sucking insects has been challenging, and alternative control methods are constantly being sought. Visual traps have long been used to monitor and control pests. Colored sticky cards are widely used for diurnal pests, but their effects are influenced by environmental light conditions. Artificial light traps are mostly used for nocturnal pests. Here, we explored and evaluated light-emitting diode (LED) traps for the monitoring and control of small diurnal sucking insects using greenhouse tests targeting the greenhouse whitefly *Trialeurodes vaporariorum*. We tested the trapping efficacy of the LED water pan trap, assessed the most attractive LED light and analyzed its efficacy under different weather conditions. The results showed that the LED water pan trap was too inefficient to be useful. Green LEDs were more attractive than yellow LEDs, UV LEDs and green-UV combinations. Regardless of sunny or cloudy conditions, the green LED trap caught more than twice the number of whiteflies than the yellow sticky card alone under summer shading conditions. Our study suggests that LED traps have a significant field application value in whitefly mass trapping and may also be efficient for other diurnal insects. The design of LED traps specific for diurnal insects is discussed.

## 1. Introduction

Due to their small size and their difficulty to detect, in addition to their short life cycle, high fecundity, and outbreak propensity, some small sucking insects, e.g., whiteflies, aphids, and thrips, have become major pests on many agricultural crops worldwide, causing serious economic losses each year. These pests, not only cause direct damage to plant tissues, but also transmit plant viruses and facilitate infections through bacteria and fungi. Furthermore, they excrete sticky honeydew, which contaminates the plant surface and serves as a growth substrate for pathogens [1]. Chemical insecticides have been the major control strategy; however, the control efficacy is not often satisfactory. With their small size, these pests easily avoid insecticide exposure when the application is not thorough. Due to the serosity produced by the pest, the immobile stages of whiteflies may avoid contact with chemical insecticides [2]. Therefore, only systematic agents are highly efficient against these sucking pests. Unfortunately, these pests easily develop resistance to repeatedly applied insecticides [3]. Thus, efforts are ongoing to seek new, efficient alternative methods in managing these pests.

Some olfactory and visual cues are attractive to insects. Plant volatile chemicals may facilitate short-distance orientation to host plants. Sex pheromones and aggregation pheromones are responsible for conspecific chemical communication and lead to calling behavior and conspecific aggregation, respectively. Visual cues play important roles in host plant location and migration navigation. All these attractive elements could be used alone, or combined with insect traps, and serve as physical control tactics.

Insects display phototactic behaviors. They can be attracted to specific light sources depending on wavelength and intensity [4,5]. Based on this attractive phototactic behavior, various visual traps have been developed to monitor or mass trap the pest population and combined with other control tactics to control the population. Colored sticky cards have been widely used in greenhouses, especially for the mass trapping of these small diurnal insects [6]. However, their efficacy is greatly affected by ambient light conditions because of varied color intensity and, hence, attractiveness [7,8]. Artificial light sources have been used in insect traps for many years to attract insects, but they are usually applied to monitor and control nocturnal pests [5]. It is well-known that the brightness and intensity of solar radiation are far more efficient than those of artificial lights, especially on sunny days, providing approximately 100,000 lumens per square meter (lx) at the Earth’s surface [9]. Whether, and to what extent, artificial lights could be effective in trapping these diurnal insects remain debatable.

To our knowledge, no efficient artificial light traps targeting these small diurnal sucking pests have been commercialized yet. Light-emitting diodes (LEDs), a recently developed semiconductor light source, could be a promising light source for insect traps. LEDs have many outstanding advantages, e.g., high luminous efficiency, low electricity consumption, small size, light weight, long lifespan, low heat emission and environmental friendliness [10]. Furthermore, their selective wavelength and intensity enable them to be specifically designed for target pests, reducing harm to beneficial and neutral insects to some extent. Some studies have shown that LED lights served as attractants [11,12,13,14,15] and increased the trapping efficacy of many insects, including greenhouse whiteflies, tobacco whiteflies, sweet potato whiteflies, thrips, and fungus gnats. However, most studies have been laboratory or confined cage studies, and field studies are still quite limited [16,17,18,19]. In this study, we used the greenhouse whitefly *Trialeurodes vaporariorum* (Westwood), an important pest worldwide, as a target insect to explore and evaluate the application potential of LED lights for the mass trapping of small diurnal insects in a greenhouse field test. The results of this study will provide valuable information for the development of a commercial highly efficient LED trap for the control of the greenhouse whitefly and will also be referential for the application of LED traps for other small diurnal insects.

A killing device and an attractive light source constitute the two major parts of a light trap. Regarding killing devices, the water pan trap is the simplest, least expensive and most commonly used among ordinary farmers. Water pan traps have been used to forecast outbreaks or mass trap aphids, thrips, etc. [5,20]. In our previous study, the LED water pan trap was highly efficient in catching fungal gnats in a dark mushroom house. How effective is it in catching the greenhouse whitefly? Does it have application value in the mass trapping of whiteflies? These are the primary questions that we hope to answer.

Regarding attractive light sources, many aphids and greenhouse whiteflies are attracted to targets that reflect or transmit light in the green-yellow range of the spectrum (520–610 nm), with yellow being the most attractive [8,21,22]. Many experiments with monochromatic lights have shown that adult insects have a peak response to green lights between 520–560 nm [14,23,24]. This shift in visual color attractiveness can be explained by the “opponent mechanism”. Aphids possess two to three classes of photoreceptors that elicit either, a direct positive response or a negative response, and achieve color detection by comparing and integrating inputs from different spectra [23]. Recently, blue-green opponency and trichromatic vision were proven in the greenhouse whitefly [25]. The receptor peaks in the greenhouse whitefly were estimated to be approximately 510–530 nm (green), 480–490 nm (blue) and 340–370 nm (UV) [23,25,26,27]. Regarding the “settling response”, the greatest attraction was achieved by green LEDs and inhibited by blue LEDs [26]. The yellow target had a reflectance spectrum with little, or no, reflectance in the blue violet spectrum [22], and the highest green/blue ratio led to the most attraction to yellow. “Migratory behavior” was elicited the most by ultraviolet (UV) light [25,27,28,29]. UV light was attractive to the greenhouse whitefly, especially in the dark, but it was less attractive than the green-yellow spectrum [8,13,16]. In a choice cage test, adult greenhouse whiteflies showed a preference for the UV-green LED complex over the green LED alone [14]. Most previous cage studies of LED traps used green and UV LEDs in their tests [13,14,16,17,19]. The most sensitive behavioral spectrum is not always the most sensitive light receptor spectrum [25]; it is the integration of visual stimuli inputs in the central nervous system that results in the peculiar phototactic response exhibited by the species [8]. The landing response is substantially influenced by a variety of factors, in addition to light wavelengths, including light intensity, the contrast between light source intensity and color to that of ambient light, and the state or physiology of the insects [5,7]. Laboratory and confined cage studies may produce bias compared with natural field environments. Therefore, field studies are necessary to identify the most attractive spectrum and evaluate the trapping efficacy. In this study, we aimed to determine the most attractive light to the greenhouse whitefly by comparing the trapping efficacy of green, yellow, and UV LEDs and their combinations under natural greenhouse conditions.

Finally, considering the most important environmental factor, solar radiation, in color/light trapping efficacy, we determined how much the trapping efficacy would be improved by LEDs under sunny and cloudy weather conditions.

In summary, in this study, we tested the trapping efficacy of the LED water pan trap for *T. vaporariorum* to determine whether this inexpensive trap could be used to mass trap the greenhouse whitefly. Then, we compared the attractiveness of green, yellow, and UV LED lights and their combinations to *T. vaporariorum* to identify the most attractive LED light for trapping. Finally, we analyzed the potentiation effect of green LEDs under different weather conditions. This study will provide valuable information about the field application potential of LED insect traps for small diurnal pests. Further suggestions for the design and development of LED traps, specific for small diurnal insects, are discussed.

## 2. Materials and Methods

### 2.1. Study Site and Insect Traps

The current study was carried out between 14 May and 29 August 2019 at Harmony Farm in the city of Erdos, Inner Mongolia Autonomous Region, China (grid-reference: 39°, 17′N; 109°, 73′E).

**Figure 1:**
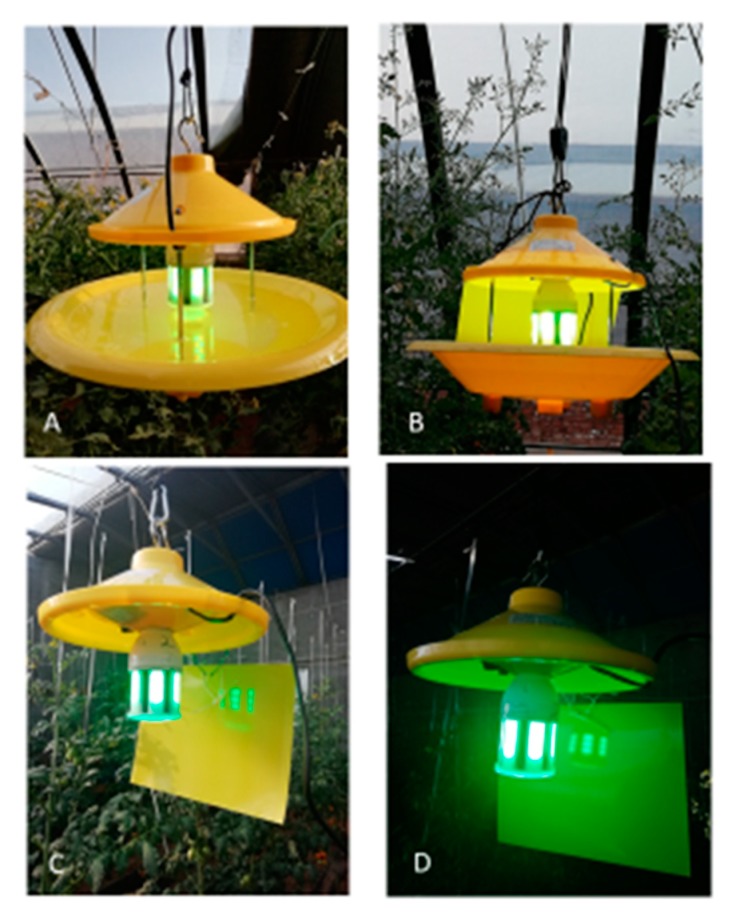
Light emitting diode (LED) traps used in this study. (**A**) LED water pan trap; (**B**) LED water pan trap supplemented with a yellow sticky card; (**C**) LED lit yellow sticky card during the daytime; (**D**) LED lit yellow sticky card during the nighttime.

The LED traps used in this study were CD-IL400-8KIG1 traps, with a rated power of 8 W and an output voltage of 6–11 V (DC). The LED lamps were arranged in a corncob shape consisting of 6 light beams. The wavelengths of the LED light used in this study were as follows: Green light, 525 ± 5 nm; yellow light, 585 ± 5 nm; and UV light, 365 ± 5 nm. Both LED traps and LED lamps were provided by Changchun Cedar Electronics Technology Co., Ltd., Changchun, China (Figure 1A). The LED lamps were on for 24 h each day during the experiment.

### 2.2. Efficacy of the LED Water Pan Trap in Monitoring the Greenhouse Whitefly Population

The experiment was carried out in a 100 × 10 m greenhouse containing tomato from 14 May to 17 June 2019; temperatures ranged from 9–30 °C and the relative humidity ranged from 40%–90%. LED water pan traps (Figure 1A) were hung in the middle of the planting rows (9 m long), with 10 alternating green and yellow traps (5 each), with a distance of 10 m between 2 traps. The bottom of the traps was hung at 1.6 m, approximately 20 cm above the tomato crown at the beginning of the experiment. Some detergents were added to the water to avoid whitefly escape. A total of 10 yellow sticky cards were placed at the middle of the two adjacent LED traps, with a distance to each LED trap of 5 m, perpendicular to the tomato row and at a similar height as the LED lamp. The numbers of greenhouse whiteflies trapped in the water pan of the LED traps and yellow sticky cards were checked on 17 June. Additionally, the number of whiteflies present on tomato leaves was checked. The whole greenhouse was divided into 3 blocks; 10 plants were randomly checked in each block, and 5 leaves from the top 3-7 branches of each plant were randomly checked.

### 2.3. Pairwise Comparison of the Trapping Efficacy of the Water Pan and Yellow Sticky Card in One LED Trap

The experiment was carried out in an intelligent greenhouse containing tomato planting of 20 × 50 m, with a temperature of 13–28 °C and relative humidity of 50%–90%. To directly compare the trapping efficacy of the water pan and yellow sticky card for greenhouse whiteflies, 4 green LED water pan traps equipped with a yellow sticky card (Figure 1B) perpendicular to the rows were evenly distributed in the middle row (50 m long), with a 10 m distance between two traps. The number of whiteflies caught in the water pan or on the yellow sticky card in each trap was checked separately each morning for 3 days.

### 2.4. Effects of Different Sides and Positions of the LED Lamp on the Trapping Efficacy of the Yellow Sticky Card

These experiments were carried out in the intelligent greenhouse. Starting in July, the shelf of the greenhouse was shaded from 10 am to 5 pm on sunny days. The temperature ranged from 15–28 °C, and the relative humidity ranged from 50%–90% during the experiment. The tomatoes (20 × 50 m) were planted in a high photosynthetic efficiency pattern in rows (50 m long) in the direction of magnetic south by west 15°. All yellow sticky cards were hung perpendicular to the rows and at the top of the tomato plants. 

In order to determine the different catch efficacies of the sunward sides (southward side) and dark sides (northward side) of the yellow sticky cards, 4 yellow sticky cards were evenly distributed in the middle row, with a 10 m distance between two traps. Considering the low whitefly population in the greenhouse at the time, the greenhouse whiteflies, trapped on the two sides of the yellow sticky cards, were checked every 3 days for 18 days.

Then, we determined whether the relative position of the LED lamp in relation to the yellow sticky card would affect the trapping efficacy of the LED-lit yellow sticky card traps. Green LED lamps were set at either the sunward side or the dark side of the yellow sticky card (refer to Figure 1C), each consisting of 4 traps hung alternated at two rows, with a 5-m distance to the edge of the planting and 10 m between 2 adjacent traps. The total number of greenhouse whiteflies trapped in each trap was checked every 3 days for 18 days.

### 2.5. Identifying the Most Attractive LED Light for the Greenhouse Whitefly

This experiment was conducted in a different 100×10 m greenhouse. Before the experiment, we randomly picked 68 leaves from the top 3-7 branches of 15 tomato plants to estimate the greenhouse whitefly population level. Considering both larvae and adults, 3.7 ± 6.5 (mean ± SD) greenhouse whiteflies were present on each leaf. During the experiment, the greenhouse was shaded overhead from 10 am to 5 pm on sunny days; the temperature ranged from 15–32 °C, and the relative humidity ranged from 40%–90%. The whole planting area was divided into 3 blocks, and 3 LED-lit yellow sticky card traps (refer to Figure 1C,D) with different wavelengths of LED light and 1 yellow sticky card were distributed evenly in the middle of the rows in each block. The number of greenhouse whiteflies caught by each trap was recorded each day, and then the position of each trap in a block was rotated sequentially. Each set of experiments lasted 3 days, so the position of each LED light was rotated completely in each block. In experiment 1, yellow, green, and UV lights were compared, and in experiment 2, green lights were compared with 2 green-UV combinations at ratios of 1/1 and 2/1.

### 2.6. Effects of Weather Conditions on the trapping Efficacy of the Yellow Sticky Card and Green LED-Lit Yellow Sticky Card Trap

We collected all the data, including counts from the yellow sticky card alone and the green LED-lit yellow sticky card trap, at the same time from the shaded greenhouse, as described above. Each trap had 30 replicates. The data were further divided into two groups depending on the weather conditions: sunny or cloudy. The effects of weather conditions on the trapping efficacy of each type of trap were analyzed.

### 2.7. Data Analysis

Statistical analysis was performed using Statistical Product and Service Solutions software version 19.0 (IBM, USA). Paired sample t tests were conducted to compare the number of greenhouse whiteflies captured between the water pan and the yellow sticky card and on the two sides of the yellow sticky cards. Analysis of variance (ANOVA) followed by the least significant difference (LSD) test for means comparisons were performed on the most attractive LED light trap data. Data were log-transformed to homogenize the variance when necessary but are presented as original data in the graphs. Other data were analyzed by independent samples *t* tests.

Data visualization was conducted with GraphPad Prism 7 (GraphPad Software, San Diego, CA, USA).

## 3. Results

### 3.1. Efficacy of the LED Water Pan Trap in Monitoring the T. vaporariorum Population

**Figure 2:**
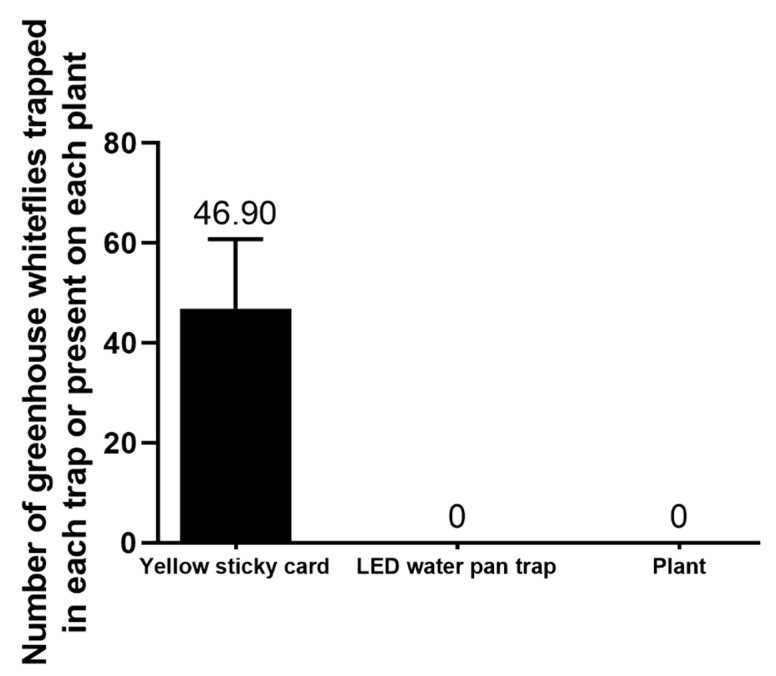
Efficacy comparison of the LED water pan trap, yellow sticky card and personal plant checking on the population monitoring of *T. vaporariorum* from 14 May to 17 June 2019. The error bar represents the standard error.

The experiment was initiated when the *T. vaporariorum* population was very low in the greenhouse and only fewer than 3 whiteflies were found occasionally on some yellow sticky cards. One month later, the average number of total whiteflies captured by each yellow sticky card reached 46.9 ± 13.9 (mean ± SEM, with a 95% confidence interval of 15.5–78.3). However, no whitefly was caught by the LED water pan trap or by plant checking (Figure 2). It can be concluded that the LED water pan trap is much less efficient than the yellow sticky card for monitoring the *T. vaporariorum* population.

### 3.2. Pairwise Comparison of the Trapping Efficacy of the Water Pan and Yellow Sticky Card in One LED Trap

**Figure 3:**
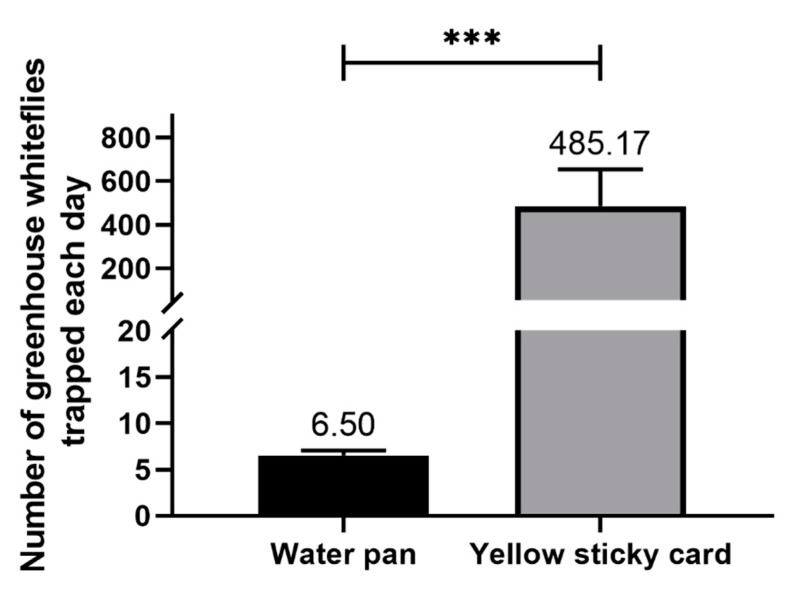
Number of *T. vaporariorum* trapped by the water pan or yellow sticky card in one LED trap. ***, Significant difference at *p* < 0.001 by the pairwise t test for the total number of captures. Error bars represent the standard error.

In one LED trap equipped with both a water pan and yellow sticky card, the number of greenhouse whiteflies trapped in the water pan was negligible (*t* = −2.827, df = 11, *p* = 0.016, Figure 3). Therefore, the LED water pan trap is not suitable for the mass trapping of greenhouse whiteflies. In the following experiment, the water pan was removed from the LED trap, and the trap was equipped with a yellow sticky card, (a LED-lit yellow sticky card), serving as the LED insect trap.

### 3.3. Effects of Different Sides and Positions of the LED Lamp on the Trapping Efficacy of Yellow Sticky Cards

**Figure 4:**
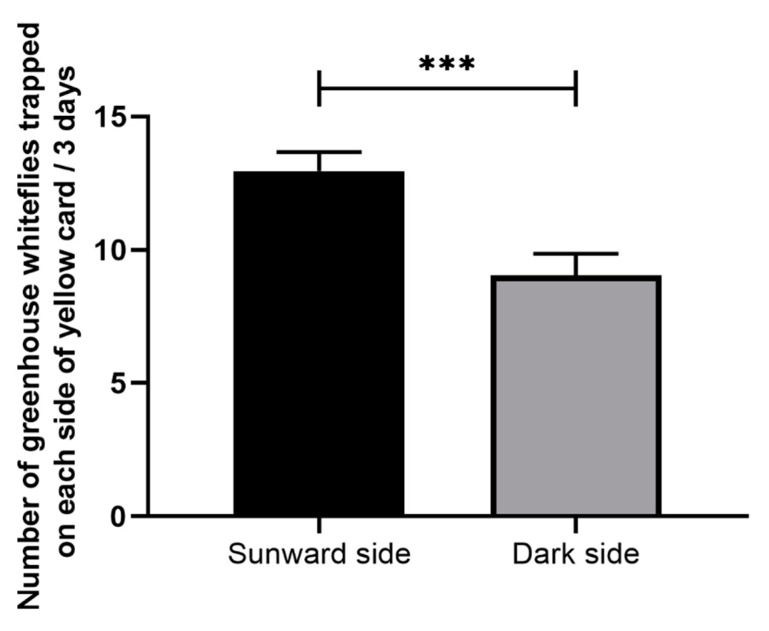
Pairwise comparison of the number of *T. vaporariorum* trapped on the sunward side and dark side of the yellow sticky card. ***, Significant difference at *p* < 0.001 by the pairwise t test. Error bars represent the standard error.

**Figure 5:**
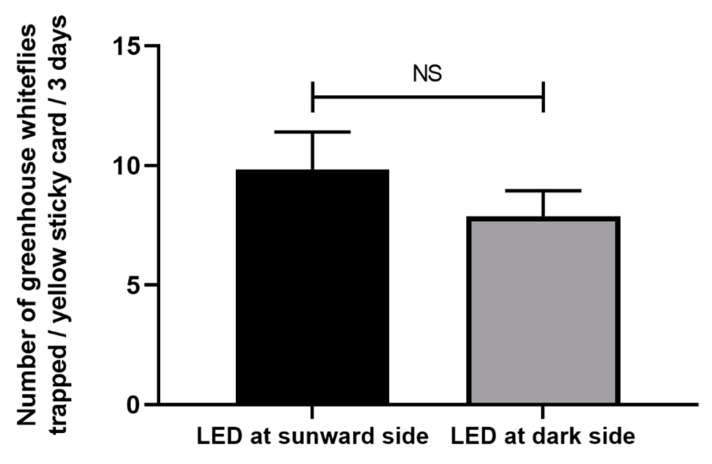
Effects of the LED lamp position (on the sunward side or the dark side of the yellow sticky card) on the capture of *T. vaporariorum* by the yellow sticky card. NS, not significant at *p* < 0.05 by the independent t test. Error bars represent the standard error.

The sunward side of the yellow sticky card caught significantly more greenhouse whiteflies than the dark side (t = 5.561, df = 23, *p* < 0.001, Figure 4). However, regardless of whether the LED lamp was placed on the sunward side or dark side of the yellow sticky card, the total number of greenhouse whiteflies trapped was not significantly different (t = 1.032, df = 46, *p* = 0.307, Figure 5).

### 3.4. Identifying the Most Attractive LED Light for the Trapping of Greenhouse Whiteflies

**Figure 6:**
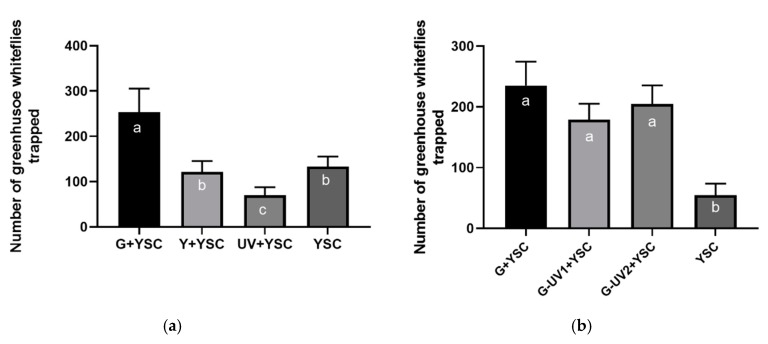
Identifying the most attractive LED light for *T. vaporariorum*. (**a**) Experiment 1, comparison between yellow, green, and UV lights; (**b**) Experiment 2, comparison between green light and its combination with different ratios of UV light. YSC, yellow sticky card; G, green LED light; Y, yellow LED light; UV, ultraviolet LED light; G-UV1, green and UV LED light ratio 2:1; G-UV2, green and UV LED light ratio 1:1. The different letters within each column indicate significant differences at *p* < 0.05 by the LSD test. Error bars represent the standard error.

In experiment 1, ANOVA suggested significant differences between the traps (F_3,32_ = 8.450, *p* < 0.001). Further analysis (by the LSD test) indicated that the yellow sticky card supplemented with a green LED light caught significantly more greenhouse whiteflies than the other 3 traps. The yellow LED light showed no significant effect on the trapping efficacy of the yellow sticky card, and the number of greenhouse whiteflies caught by the yellow sticky card supplemented with the UV LED light was the least efficient and caught significantly less whiteflies than the other 3 traps (Figure 6a).

In experiment 2, ANOVA suggested a significant difference between the traps (F_3,32_ = 7.125, *p* < 0.001). Further analysis (by the LSD test) indicated that the presence of a UV LED light had no significant effect on the trapping efficacy of the green LED light. All 3 yellow sticky card traps equipped with a green LED light caught significantly more greenhouse whiteflies than the yellow sticky card alone (Figure 6b).

### 3.5. Effects of Weather Conditions on the Trapping Efficacy of Yellow Sticky Cards or Green LED-Lit Yellow Sticky Card Traps

**Figure 7:**
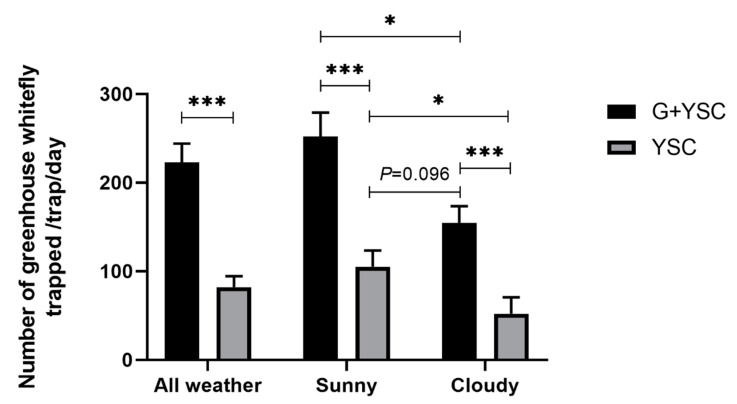
Effects of weather conditions on the trapping efficacy of the yellow sticky card or green LED-lit yellow sticky card trap. YSC, yellow sticky card; G, green LED light; *, significant difference at *p* < 0.05 by the independent t test; ***, significant difference at *p* < 0.001 by the independent t test. Error bars represent the standard error.

The results showed that regardless of the weather condition, the trapping efficacy of the green LED-lit yellow sticky card trap was significantly higher than that of the yellow sticky card alone (for sunny days, *t* = 4.321, df = 36, *p* < 0.001; for cloudy days, *t* = 4.928, df = 20, *p* < 0.001; for all days, *t* = 5.764, df = 46.524, *p* < 0.001). On the other hand, both traps caught significantly more greenhouse whiteflies on sunny days than on cloudy days (for yellow sticky cards, *t* = 2.468, df = 25.745, *p* = 0.021; for LED traps, *t* = 2.266, df = 28, *p* = 0.031). Furthermore, the green LED-lit yellow sticky card trapped even more whiteflies on cloudy days than the yellow sticky card alone on sunny days (*t* = −1.730, df = 24, *p* = 0.096, Figure 7).

## 4. Discussion

Although, our previous study showed that the LED water pan trap was quite effective in trapping small fungus gnats under mushroom cultivation conditions, it was useless in catching greenhouse whiteflies. We presume that the failure of the LED water pan trap is due to these insects are active during the day. In the dark, the reflection of the LED light on the water is very clear and bright, which may attract more insects to fly into the water. However, even on a cloudy day in the greenhouse, the reflection of the LED light on the water was not clear, and its brightness was much less than that of LED light itself during the daytime. Therefore, even if greenhouse whiteflies are attracted to LED lights, they are not encouraged to fly into the water. Although yellow water pan traps have been used to capture aphids and whiteflies [5,20], their failure in LED traps suggests that the attractiveness of LED light is much greater than that of yellow water pans. It is rational to speculate that the LED water pan trap is also not suitable for the trapping of other small diurnal insects. LED traps should be equipped with other types of killing devices when targeting these small diurnal insects; fan driving suction traps may be a better choice. With suction force, these small insects have no opportunity to escape when they reach the light trap. On the other hand, this study also showed that the yellow sticky card is a simple and effective way to monitor the greenhouse whitefly population; it is much earlier than direct visual counts on plants [6].

With the failure of the LED water pan trap, we evaluated the potential trapping efficacy of LED light for the greenhouse whitefly by adding a yellow sticky card to the LED trap. As they are inexpensive and effective, yellow sticky cards have been widely used in greenhouses. Thus, a yellow sticky card alone was used as a control. If an LED-lit yellow sticky card trap catches significantly more whiteflies than a yellow sticky card alone, it will have application value in mass trapping. This field evaluation was carried out with the experiments of identifying the most attractive LED light spectrum to greenhouse whiteflies.

Light is an important cue for insect orientation and host location [30,31]. The peak sensitivity of greenhouse whitefly optical receptors occurred at 525 nm around green light [14,25,26,27]. The “opponent mechanism” of positive input from the green receptor coupled with negative input from the blue or UV receptor enables greenhouse whiteflies or aphids to differentiate various colors, and thus, respond differently [23,25]. A yellow card, arising from the extremely high ratio of green/blue reflection, is the most attractive to greenhouse whiteflies and aphids [22]. In the experiment containing the yellow sticky card alone, and the yellow card equipped with green, yellow and/or UV LED lights, only the yellow sticky cards equipped with green LED lights caught significantly more greenhouse whiteflies than the yellow sticky cards alone. This result is consistent with the most sensitive spectrum of the optical receptors. Our experimental system consisted of a LED light and a yellow sticky card together. Therefore, it is the green LED-lit yellow sticky card that showed the highest trapping efficacy. 

Aphids and whiteflies locate host plants via the contrast between the soil background and the color reflection from the plant foliage [26,30,32]. A high contrast between the color of the card and the background increase the attractiveness level [33,34]. Based on field observations, green LED lights produced a bright green reflection on the yellow sticky card (cell phone picture in Figure 1C was overexposed), while the reflections on the yellow sticky cards from yellow and UV LEDs were not obvious. In addition to optical sensitivity, a high reflection or contrast of green LED light against a yellow sticky card, in addition to the high contrast of the yellow sticky card against green plants and soil, may also be responsible for the high attraction to the green LED-yellow sticky card complex. Therefore, due to visual sensitivity and visuality, green LED light significantly increased the attractiveness of yellow sticky cards.

UV light is important for orientation, navigation and host finding [1,28]. Many insects prefer to move toward environments with a higher intensity of UV light [35,36]. However, aphids and whiteflies seem to be repelled by high-intensity UV light [37]. A tunnel test carried out in the dark showed that UV light is attractive to greenhouse whiteflies [13]. However, in our field test, the yellow sticky card equipped with UV LED lights caught the least number of greenhouse whiteflies in a 24 h observation period. This weak attractiveness of the UV LED light was consistent with the results of some cage tests [14,16]. Yellow sticky cards alone were more attractive to greenhouse whiteflies than those supplemented with UV LED lights, both during the day and at night time. In a choice cage test with green LEDs either, alone or plus UV LEDs, improved capture occurred with the green plus UV light traps [14]. However, the increased attractiveness of UV to green LED lights was not observed in our natural greenhouse test. Greenhouse whitefly flight is affected by many environmental factors, such as temperature and plant vegetative conditions, as well as by the physiological state of the insect [37]. During long-distance flights, such as dispersal and migration flights, insects are usually attracted to the UV spectrum [1,38,39]. Once these flights are terminated, insect attraction to UV light decreases, and attraction to the yellow-green light spectrum for the location of potential host plants increases [26,30,31,39,40]. In the tomato growing season, if the whitefly population is not too high to support their growth and fecundity, most of their flies in the greenhouse will be only short distances. During short-distance flights, whiteflies are more attracted to the green-yellow spectrum than to UV light. We are not sure whether mechanical manipulations, such as whitefly releases, elicit an increased tendency for flight, and whether this active flying will increase the attractiveness of UV lights significantly, as each observation time lasted only 1.5 h in cage tests [14]. Natural field performance may be different from that in confined experimental settings. In our field test, disturbance of the greenhouse whitefly was neglectable, and all data were collected after 24 h. No potentiation in the attractiveness of green LEDs was observed by the addition of UV LEDs. Our results suggest that for greenhouse whiteflies, green LEDs are the most attractive lights, UV LEDs are the least attractive lights, and UV lights do not increase the trapping efficacy of green LEDs.

On sunny days, strong reflections make yellow sticky cards very bright, and they will catch many more greenhouse whiteflies than that on cloudy days. During our experiment, the greenhouse shelf was shaded from 10 am to 5 pm on sunny days, and the total captures of greenhouse whiteflies by both, the yellow sticky card alone and the yellow sticky card, supplemented with green LED lights were significantly higher on sunny days than that on cloudy days, suggesting the great power of solar radiation. On the other hand, green LEDs increased the trapping efficacy of yellow sticky cards by more than one-fold under both, sunny and cloudy conditions, indicating the power of green LED lights. Furthermore, the number of whiteflies trapped by the green LED-yellow sticky card trap on cloudy days was even higher than that of the yellow sticky card alone on sunny days. Therefore, applying green LED lights to yellow sticky card traps effectively increased the trapping efficacy for greenhouse whiteflies. Note that these results were derived from a shaded greenhouse. Further tests are required to understand whether LED lights are as efficient as in this study, in circumstances where the greenhouse is not shaded, which occurs during all months of the year except July and August. For solar greenhouses, outbreaks of greenhouse whiteflies usually occur in July and August, but for intelligent greenhouses, outbreaks of greenhouse whiteflies usually occur much earlier and last longer periods because of the well manipulated temperatures.

To better utilize both solar radiation and LED lights, we suggest that both yellow card and green LED be included in the design of LED insect traps as attractive elements for greenhouse whiteflies. The trapping efficacy could be increased further with better organization, for example, optimized intensity and spectrum of LED lamp, and yellow cards position at the greatest utilization of solar radiation. Considering the similar vision mechanism and yellow preference in aphids, the specifically designed LED trap for greenhouse whiteflies may also be effective for winged aphids. The basic design of the LED insect trap could be the same for all small diurnal pests, except for the light attractant portion of the color card and LED light, which could be tailored to the target pest. The color card and LED light could be changed according to the target pest. This ensures both a wide application range and target specificity. Target specificity should be another developmental direction for insect traps, as they are more ecofriendly and beneficial to biodiversity preservation and sustained pest management than general insect traps.

## 5. Conclusions

The purpose of this study was to explore and evaluate the field application of LED insect traps for the monitoring and control of small diurnal sucking pests by using greenhouse whiteflies, *T. vaporariorum*, as an example. First, we tested the trapping efficacy of the LED water pan trap for *T. vaporariorum* and proved that it has no application value. Then, we compared the attractiveness of green, yellow and UV LED lights and their combinations to *T. vaporariorum* and found that green LEDs were the most attractive light and addition of UV LEDs did not increase the attractiveness of green LEDs. Finally, we analyzed the effects of green LEDs under different weather conditions. It was found that the number of captures on sunny days was much higher than that on cloudy days. Regardless of the weather (sunny or cloudy), the green LEDs increased the number of captures by more than one-fold compared with the yellow sticky card alone during the summer shading operation. Therefore, we suggest that both colored card and LED light should be included in the design of an LED insect trap to achieve the greatest attraction. Our study suggests that the LED trap has significant field application value in the mass trapping of greenhouse whiteflies and may also be efficient for the trapping of other diurnal insects if equipped with a species-specific attractive color card and LED light.

## 6. Patents

Based on this study, we developed the patent “A LED insect trap for small diurnal insects” (Application number: China 2019221589118).
